# Highlights on Novel Technologies for the Detection of Antibodies to Ro60, Ro52, and SS-B

**DOI:** 10.1155/2013/978202

**Published:** 2013-11-27

**Authors:** M. Infantino, C. Bentow, A. Seaman, M. Benucci, F. Atzeni, P. Sarzi-Puttini, B. Olivito, F. Meacci, M. Manfredi, M. Mahler

**Affiliations:** ^1^Immunology and Allergology Laboratory Unit, Azienda Sanitaria di Firenze, San Giovanni di Dio Hospital, Florence, Italy; ^2^Department of Research, INOVA Diagnostics Inc., 9900 Old Grove Road, San Diego, CA 92131-1638, USA; ^3^Rheumatology Unit, Department of Internal Medicine, San Giovanni di Dio Hospital, Azienda Sanitaria di Firenze, Florence, Italy; ^4^Rheumatology Unit, L. Sacco University Hospital, Milan, Italy

## Abstract

*Objective*. We aimed to compare a chemiluminescent immunoassay (CIA, QUANTA Flash) on BIO-FLASH with a multiplex flow immunoassay (MFI) on BioPlex 2200 for the detection of antibodies to Ro60, Ro52, and SS-B. *Methods*. The study included 241 samples, from patients suffering from systemic autoimmune diseases (*n* = 108) as well as disease controls (*n* = 133). All samples were tested for anti-Ro52, anti-Ro60, and anti-SS-B (La) antibodies on QUANTA Flash (INOVA Diagnostics, San Diego, USA) and BioPlex 2200 (Bio-Rad Laboratories Inc., Hercules, USA). Discrepant samples were tested by two independent methods: BlueDot/ANA and QUANTRIX Microarray (both D-tek, Belgium). *Results*. The overall qualitative agreements were 95.4% (95% confidence interval, CI 92.0–97.7%) for anti-Ro52, 98.8% (95% CI 96.4–99.7%) for anti-Ro60, and 91.7% (95% CI 87.5–94.9%) for anti-SS-B antibodies. There were 34 discrepant samples among all assays (20 anti-SS-B, 11 anti-Ro52, 3 anti-Ro60). 30/33 of retested samples (by D-tek dot blot) agreed with the QUANTA Flash results. Similar findings were obtained with QUANTRIX Microarray kit. *Conclusion*. QUANTA Flash and BioPlex 2200 show good qualitative agreement. The clinical performances were similar for anti-Ro52 and anti-Ro60 autoantibodies while differences were observed for anti-SS-B (La) antibodies.

## 1. Introduction

Autoantibodies targeting extractable nuclear antigens (ENA) are hallmarks in the diagnosis of systemic autoimmune rheumatic disease (SARD) such as systemic lupus erythematosus (SLE), Sjögren's syndrome (SjS), systemic sclerosis (SSc), polymyositis/dermatomyositis (PM/DM), and mixed connective tissue disease (MCTD). In addition, anti-ENA antibodies can be detected in undifferentiated connective tissue disease (UCTD) [1]. The primary antigenic targets of anti-ENA antibodies are U1-ribonucleoproteins (RNP), Sm (Smith antigen) [2], Scl-70 (topoisomerase I) [3], Jo-1, Ro60 (SS-A) [4], Ro52 (TRIM21) [4], and SS-B (La) [1]. Not all of those antibodies are specific for a particular disease but are useful to help ruling in or out SARD [1]. Among the most common autoantibodies are those to Ro52, Ro60 and SS-B [1]. Historically, anti-Ro52 and anti-Ro60 antibodies combined have been detected and reported [4]. However, recent data suggested that both the cellular function of the two proteins and the disease association of anti-Ro52 and anti-Ro60 antibodies are significantly different [1, 4, 5]. In addition, about 20% of those antibodies can be missed when tested using a blend of the two antigens [4]. Besides the diagnostic value of antibodies to Ro52, Ro60, and SS-B, it has been shown that those antibody specificities can precede the clinical onset of SLE for many years [6]. In addition, antibodies to the three antigens characterize a subpopulation of SLE patients that are clinically different from other SLE patients [7]. Several assays have been developed and used for the detection of anti-ENA antibodies including ELISA, line immunoassays (LIA) [8], multiplex flow immunoassay (MFI) mostly referred to as addressable laser bead assays (ALBIA) [9–12] and protein arrays [13]. In recent years, the chemiluminescence technology, which has been used for clinical chemistry for more than 10 years, has been applied for autoantibody testing [14, 15]. The objective of the present study was to analyze the performance of novel chemiluminescent immunoassays (CIA, QUANTA Flash) on BIO-FLASH, a rapid-response chemiluminescent analyzer in comparison with multiplex flow immunoassay on BioPlex 2200 system for the detection of antibodies to Ro60, Ro52, and SS-B. Additionally, the clinical utility of antibody titer and multiple positivity [16] were analyzed.

## 2. Materials and Methods

### 2.1. Sera

The study included 241 samples from patients suffering from SARD (*n* = 108) as well as disease controls (*n* = 133). All samples were tested for anti-Ro52, anti-Ro60, and anti-SS-B antibodies by QUANTA Flash (INOVA Diagnostics, San Diego, USA) and BioPlex 2200 (Bio-Rad Laboratories Inc., Hercules, USA). Discrepant samples were tested by two independent methods: BlueDot/ANA and QUANTRIX Microarray (both D-tek, Belgium). The diagnoses were established as described before [17] or according to the standard disease criteria.

This study meets and is in compliance with all ethical standards in medicine, and informed consent was obtained from all patients according to the Declaration of Helsinki.

### 2.2. QUANTA Flash Assays

The QUANTA Flash assays (INOVA Diagnostics Inc., San Diego, CA, USA) are novel CIAs that are used on the BIO-FLASH instrument (Biokit S.A., Barcelona, Spain), fitted with a luminometer, as well as all the hardware and liquid handling accessories necessary to fully automate the assay. The principle of the BIO-FLASH system has recently been described [14, 15]. The QUANTA Flash assays for this study were developed using recombinant antigens (INOVA Diagnostics, see Table 1) coated onto paramagnetic beads. Prior to use, the lyophilized beads are resuspended using the resuspension buffer. A patient serum sample is prediluted with the BIO-FLASH sample buffer in a small disposable plastic cuvette. Small amounts of the diluted patient serum, the beads, and the assay buffer are all combined into a second cuvette, mixed, and then incubated for 9.5 minutes at 37°C. The magnetized beads are sedimented using a strong magnet in the washing station and washed several times followed by addition of isoluminol conjugated anti-human IgG and again incubated 9.5 minutes at 37°C. The magnetized beads are sedimented and washed repeatedly. The isoluminol conjugate is oxidized when sodium hydroxide solution and peroxide solutions (“Triggers”) are added to the cuvette, and the flash of light produced from this reaction is measured as Relative Light Units (RLUs) by the BIO-FLASH optical system. The RLUs are proportional to the amount of isoluminol conjugate that is bound to the human IgG, which is in turn proportional to the amount of autoantibodies bound to the antigen on the beads.

### 2.3. BioPlex 2200

BioPlex 2200 (Bio-Rad, Hercules, CA) system is an automated analyzer that uses multiplex bead technology (Luminex, Austin, TX, US) to simultaneously detect antibodies to several antigens in a single tube. The BioPlex 2200 ANA Screen is intended for the qualitative screening of ANA, the quantitative detection of antibody to dsDNA, and the semiquantitative detection of ten separate antibodies (Chromatin, Ribosomal P, SS-A, SS-B, Sm, SmRNP, RNP, Scl-70, Jo-1, and Centromere B) [10, 11] in human serum and/or EDTA or heparinized plasma. The test system is used as an aid in the diagnosis of SARD. The system reports anti-Ro52 and anti-Ro60 antibodies as individual results outside the United States and the combined result as anti-SS-A in the United States due to lack of 510 K clearance by the Food and Drug Administration (FDA) of the anti-Ro52 antibody assay. Characteristics of the assay are summarized in Table 1.

### 2.4. QUANTRIX and Dot Blot

ANA12 IgG BlueDot (ANA12D+DFS70) and ANA PROFILE 25 Ag DOT (Code: AD ANA25DBD) for BlueDiver Instrument (both D-tek, Belgium) were used as comparator methods on discrepant samples. ANA12 IgG BlueDot contains the antigens: Nucleosome, Sm, RNP (68 kD/A/C), Ro60, Ro52, SSB(La), Jo-1, Scl-70, CENP-A/B, PCNA, Ribosome P(P0), and DFS70. For this study, only anti-Ro60, anti-Ro52, and anti-SS-B antibodies were used. The test procedure followed the instruction for use (see http://www.d-tek.be/). ANA PROFILE 25 Ag DOT contains the antigens: Nucleosome, dsDNA, Histones, Sm, RNP, Sm/RNP, Ro60, Ro52, SSB(La), Scl-70, Ku, PM/Scl-100, Mi-2, Jo-1, PL-7, PL-12, SRP, Ribosome P(P0), CENP-A/B, PCNA, sp100, gp210, M2 recombinant, M2/nPDC, and f-actin. For this study, only Ro60, Ro52, and SS-B were used.

### 2.5. Statistical Analyses

The data were statistically evaluated using the Analyse-it software (Version 1.62; Analyse-it Software, Ltd., Leeds, UK). Chi-square, Spearman's correlation, and Cohen's *kappa* agreement test were carried out to analyze the agreement between portions, and *P* values < 0.05 were considered significant. Receiver operating characteristics (ROC) analysis was used to analyze the discriminatory ability of different immunoassays. Cluster analysis was used to illustrate the relationship between different assays [18] and to display the reactivity pattern of the patients. Hierarchical clustering was performed using average linkage clustering where patient correlation was performed uncentered and the reactivities uncentered.

## 3. Results

### 3.1. Correlation between QUANTA Flash and BioPlex 2200

**Figure 1:**
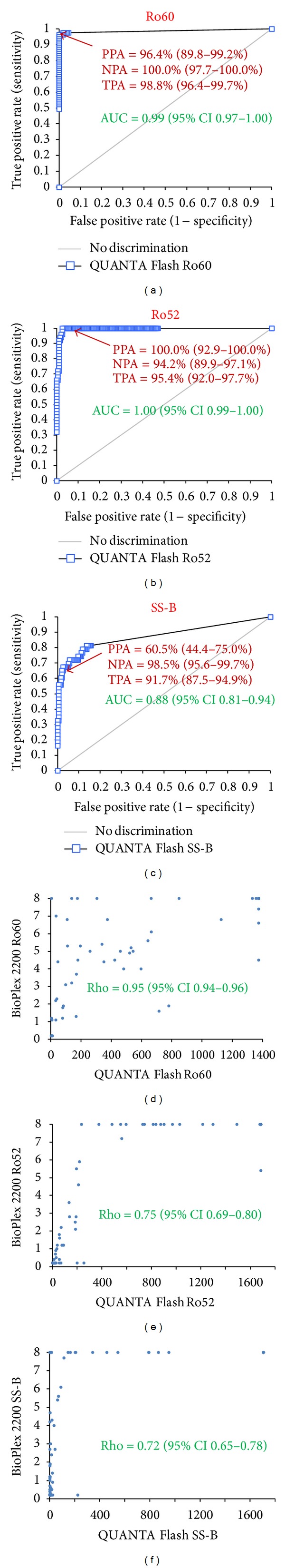
Comparison of QUANTA Flash with BioPlex 2200 as comparative method. Receiver operating characteristic (ROC) curves are shown in (a)–(c) and Spearman's correlation diagrams in (d)–(f). Note: A significant portion of positive samples are above the analytical measuring range of BioPlex which biases the Spearman *rho* values. PPA/NPA/TPA: Positive/Negative/Total % agreement. AUC: area under the curve.

The overall qualitative agreements between QUANTA Flash and BioPlex 2200 were 95.4% (95% confidence interval, CI 92.0–97.7%) for anti-Ro52, 98.8% (95% CI 96.4–99.7%) for anti-Ro60, and 91.7% (95% CI 87.5–94.9%) for anti-SS-B antibodies (Table 2). Using ROC analyses with the BioPlex 2200 results as the comparator, excellent agreement was found for anti-Ro60, good for anti-Ro52, and moderate for anti-SS-B antibodies. Areas under the curve (AUC) values were 0.99 for anti-Ro60, 1.00 for anti-Ro52, and 0.88 for anti-SS-B antibodies (see Figure 1). Additionally good quantitative agreements were observed. The Spearman rho values were: 0.95 (95% 0.94–0.96) for anti-Ro60, 0.75 (95% 0.69–0.80) for anti-Ro52, and 0.72 (95% 0.65–0.78) for anti-SS-B antibodies.

### 3.2. Clinical Performance of QUANTA Flash and BioPlex 2200

**Figure 2:**
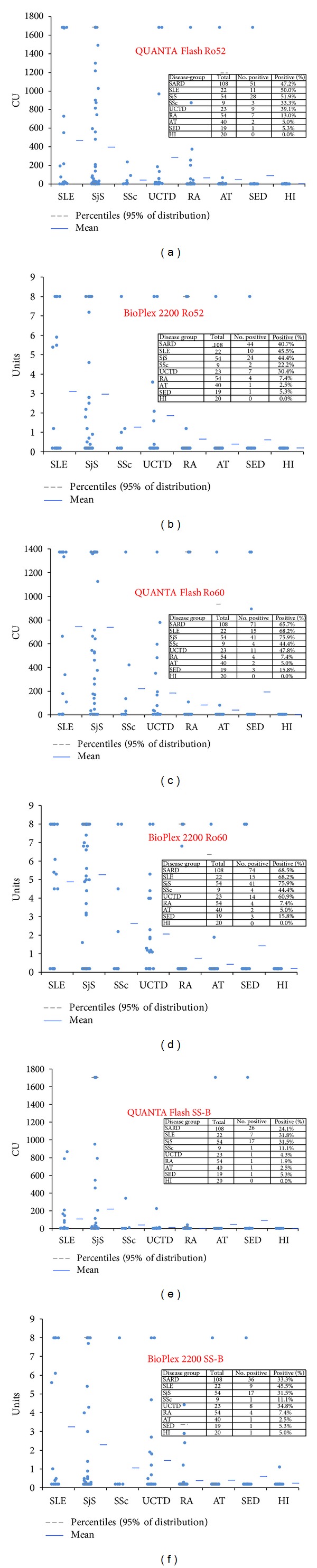
Prevalence and titers of anti-Ro60, Ro52, and SS-B antibodies. Results for anti-Ro60 are shown in (a) and (b), for anti-Ro52 in (c) and (d), and for anti-SS-B antibodies in (e) and (f). AT: Atopic dermatitis, HI: healthy individuals, RA: rheumatoid arthritis, SED: suspected eye disease, SLE: systemic lupus erythematosus, SjS: Sjögren's syndrome, SSc: systemic sclerosis, UCTD: undifferentiated connective tissue disease.

**Figure 3:**
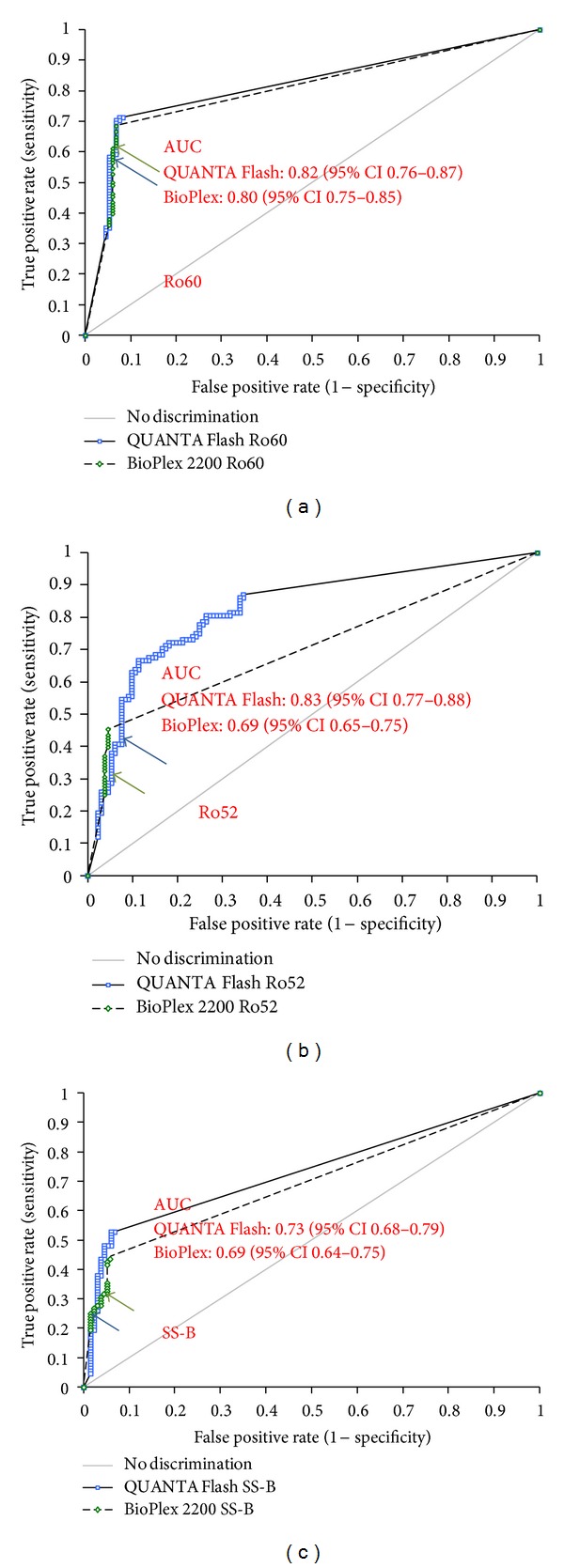
Clinical comparative ROC analysis. Results of patients with systemic autoimmune rheumatic diseases were compared with controls. Cut-off values are indicated by arrows. For sensitivity and specificity, see Table 1. Results for anti-Ro60 (a), anti-Ro52 (b), and anti-SS-B antibodies (c) are shown.

The prevalence and titers of anti-Ro52, anti-Ro60, and anti-SS-B antibodies in different cohorts using both assay methods can be found in Figure 2. Comparative ROC analyses were performed on all assays for patients with SARD compared with controls and showed similar results for QUANTA Flash and BioPlex 2200 for anti-Ro60 antibodies (see Figure 3). For anti-Ro52 antibodies the AUC value was significantly higher for QUANTA Flash compared to BioPlex 2200 (0.82 versus 0.69; *P* < 0.0001). However, the difference was found in the nonclinically relevant area of the AUC. For anti-SS-B antibodies the AUC value was slightly higher for QUANTA Flash compared to BioPlex 2200 (0.73 versus 0.69; *P* > 0.05). The sensitivities and specificities among SARD, SLE, and SjS patients are shown in Table 1.

### 3.3. Analysis of Discrepant Samples

**Figure 4:**
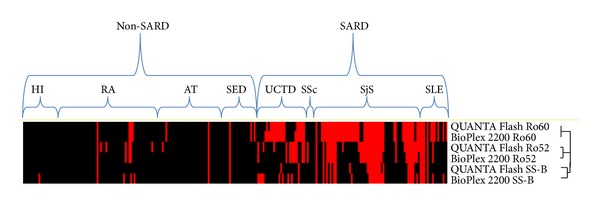
Supervised cluster analysis of the results. Supervised centered cluster analysis according to disease cohort is shown. The dendrogram shows that the Ro60 assays are closely related, the Ro52 assays are somewhat related, and the SS-B assays show significant difference. AT: atopic dermatitis; UCTD: undifferentiated connective tissue disease, RA: rheumatoid arthritis, SSc: systemic sclerosis; SED: suspected eye disease, SLE: systemic lupus erythematosus; HI: healthy individuals; SARD: systemic autoimmune rheumatic disease.

Discrepant samples (20 anti-SS-B, 11 anti-Ro52, 3 anti-Ro60) with sufficient residual volume (*n* = 33) were tested by two comparative methods. Thirty out of 33 discrepant samples among all assays (19 anti-SS-B, 11 anti-Ro52, 3 anti-Ro60) retested by D-tek dot blot agreed with the QUANTA Flash results (Table 3). Five of the 20 anti-SS-B discrepant samples were high positive on BioPlex (>8.0 units, above the AMR) and negative on QUANTA Flash, BlueDot, and QUANTRIX. Of the 17 BioPlex anti-SS-B positive/QUANTA Flash anti-SS-B negative samples 14 were from SARD and 4 from non-SARD patients (3 RA and one HI). All three QUANTA Flash anti-SS-B positive/BioPlex anti-SS-B negative samples were from SARD patients. All three were additionally anti-Ro60 antibody positive by both methods (QUANTA Flash and BioPlex).  Figure 4 depicts a cluster analysis of all methods sorted by disease group, which serves as a visual aid of the antibody prevalence and differences between methods.

### 3.4. Effect of Multiple Positivity and Titer on Likelihood of Disease

To analyze the clinical utility of autoantibody titer and multiple positivity as previously described [16], we analyzed (a) the performance characteristics at the cut-off corresponding to the highest LR+ in comparison with the recommended cut-off and (b) multiple positivity (Table 4). Double positivity provided the highest LR+ for SARD compared to the controls (both for BIO-FLASH and BioPlex 2200). Highest LR+ (12.31/16.3) was obtained with SS-B at a low cut-off of 6.3 CU (QUANTA Flash)/4.3 units (BioPlex).

## 4. Discussion

The detection of anti-ENA antibodies is important to help in the diagnosis of SARD [1]. In recent years, several new and automated methods for the detection of anti-ENA antibodies have been developed [10, 11, 14, 15]. However, the standardization of antibody assays is still not nearly accomplished, and significant variations between different assays have been reported [19]. Since reliable detection of autoantibodies is of high importance, careful verification and validation of the assay performance is mandatory. Since the prevalence of antibodies to the other antigens contained in the BioPlex 2200 ANA profile was low and did not allow for statistical evaluation, we focused on the comparison between QUANTA Flash and BioPlex 2200 assays for the detection of anti-Ro52, anti-Ro60, and anti-SS-B antibodies.

A broad range of line immunoassays (LIA) and dot-blot assays are available and are usually used to confirm previous identified autoantibodies [20, 21]. Mainly manually performed, these assays offer a simple way of multiplex testing and even automated LIAs have become available [8]. Therefore, we choose a Dot blot assay (variation of LIA) and a microarray as confirmation assays for the discrepant samples. Multiplex assays based on the Luminex technology use addressable laser beads and are therefore often referred to as ALBIA (addressable laser bead assays) or multiplex flow immunoassay (MFI) [9]. Today several commercial MFI kits are available for the detection of autoantibodies to nuclear antigens [10, 11, 22, 23]. Similar to LIAs, the number of antigens and the antigen composition significantly varies. First generation MFI showed polyreactivity which was caused by unspecific binding to the beads. Second generation showed significant reduced polyreactivity and thus higher specificity [24]. Multiplex assays are commonly used as a screening test for ANA or other autoantibodies. Additional methods have been developed for automated ANA detection [25–27]. Several publications have described protein arrays for the detection of autoantibodies to nuclear antigens [13]. However, until today those assays are not widely used in routine laboratories. The microarray used in this study showed good agreement with other methods and might represent a promising multiplex platform for autoantibody detection.

Although the QUANTA Flash and BioPlex 2200 use different sources of antigens (recombinant versus native for Ro60), the results are very similar. During the last decade, significant improvements have been made in recombinant protein technology [28]. In particular, novel strategies for the generation of recombinant Ro60 led to the availability of this antigen as a high quality recombinant protein [4]. Recombinant antigen manufacturing is more consistent and less dependent on the biological variations of the source material [28]. Therefore, the novel CIA shows similar assay performance combined with high degree of precision and consistency [15]. Despite the fact that both systems use recombinant Ro52 antigen, differences in the results were observed. Therefore, the difference might be related to the different antigen immobilization (bead chemistry) between the QUANTA Flash and the BioPlex 2200 system. However, our total percent agreement (95.4%, Table 2) is significantly better than agreements previously reported [5]. One putative reason for the significant difference between the QUANTA Flash and BioPlex 2200 results for anti-SS-B antibodies is the antigen source. The QUANTA Flash SS-B assay utilizes recombinant SS-B expressed in insect cells, whereas the BioPlex 2200 is based on native SS-B antigen [15]. Retesting of the discrepant samples using two additional methods mostly confirmed the QUANTA Flash results.

It is also important to point out that 5/17 BioPlex 2200 anti-SS-B positive samples (range 1–1.2 units) and 2/3 anti-SS-B positive samples by QUANTA Flash (range 20–25 CU) were low positive. Therefore, it is possible that a modified cut-off value would increase the agreement between BioPlex 2200 and QUANTA Flash SS-B. In the case of anti-SS-B discrepant samples, 16/20 are from SARD and 4 from “non-SARD” patients (3 RA and 1 HI). It is relevant that 3/4 “non-SARD” but only 3/16 of the SARD patients are only anti-SS-B positive. Since anti-SS-B antibodies occur in different SARD and no “gold standard” is available for the detection of anti-SS-B antibodies, it remains speculative which results are clinically correct and meaningful.

When compared to previous studies which analyzed the performance of the BioPlex 2200 system [10, 11], our data were in general agreement with the published data. In a large study on 510 healthy individuals, 0.2% (anti-Ro52), 0.6% (anti-Ro60), and 0.8% (anti-SS-B) were positive [10]. Despite the small number of HI used in our study, our data confirmed the specificity against HI. In a second study, the prevalence of antibodies to Ro52, Ro60, and SS-B was described [16]. Overall, the results were in agreement or not significant due to small patient groups. The QUANTA Flash assays have just been launched, and therefore only one paper has been published [16].

Historically autoantibody test results were mostly considered individually. Recently, several studies reported increased utility by combining autoantibody assay results and by considering antibody titers [16]. Consequently, we strived to analyze the synergy effect of combining the results of antibody testing for anti-Ro52, anti-Ro60, and anti-SS-B antibodies. Additionally, we analyze the impact of the antibody titers on the likelihood of disease. Although we found increased performance characteristics when combining results and with optimized cut-off values, the incremental value was not as pronounced as previously reported [16]. This might be explained by the strong overlap between the three antibodies we studied.

## 5. Conclusion

QUANTA Flash and BioPlex 2200 show good qualitative agreement. The clinical performances were similar for anti-Ro52 and Ro60 autoantibodies while differences were observed for anti-SSB (La) antibodies.

## Highlights

- Good agreements between QUANTA Flash and BioPlex 2200 were found for anti-Ro52 and anti-Ro60.
- Retesting of discrepant samples confirm in the majority of cases the QUANTA Flash results.
- Antibody titer and multiple positivity might provide additional value for anti-ENA testing.

## Figures and Tables

**Table 1:** Sensitivities and specificities of the different assays in different diseases.

	QUANTA Flash Ro52	BioPlex 2200 Ro52	QUANTA Flash Ro60	BioPlex 2200 Ro60	QUANTA Flash SS-B	BioPlex 2200 SS-B
Antigen source	Recombinant, insect cells	Recombinant, insect cells	Recombinant, insect cells	Native	Recombinant, insect cells	Native
Analytical measuring range (cut-off)	2.3–1685.3 CU (20 CU)	0.2–8.0 units (1.0 units)	4.9–1374.8 CU (20 CU)	0.2–8.0 units (1.0 units)	3.3–1706.8 CU (20 CU)	0.2–8.0 units (1.0 units)
Sensitivity in SARD % (95% CI)	47.2 (37.5–57.1)	40.7 (31.4–50.6)	65.7 (56.0–74.6)	68.5 (58.9–77.1)	24.1 (16.4–33.3)	33.3 (24.6–43.1)
Sensitivity in SLE % (95% CI)	50.0 (28.2–71.8)	45.5 (24.4–67.8)	68.2 (45.1–86.1)	68.2 (45.1–86.1)	31.8 (13.9–54.9)	45.5 (24.4–67.8)
Sensitivity in SjS % (95% CI)	51.9 (37.8–65.7)	44.4 (30.9–58.6)	75.9 (62.4–86.5)	75.9 (62.4–86.5)	31.5 (19.5–45.6)	31.5 (19.5–45.6)
Specificity % (95% CI)	92.5 (86.6–96.3)	95.5 (90.4–98.3)	93.2 (87.5–96.9)	93.2 (87.5–96.9)	97.7 (93.5–99.5)	94.7 (89.5–97.9)

**Table:** (a)

All patients (*n* = 241)	BioPlex 2200 Ro52			Percent agreement (95% confidence interval)
	Positive	Negative	Total	
QUANTA Flash Ro52				
Positive	50	11	61	Pos agreement = 100.0% (92.9–100.0%)
Negative	0	180	180	Neg agreement = 94.2% (89.9–97.1%)
Total	50	191	241	Total agreement = 95.4% (92.0–97.7%)
				*kappa* = 0.87 (95% CI 0.80–0.95)

**Table:** (b)

All patients (*n* = 241)	BioPlex 2200 Ro60			Percent agreement (95% confidence interval)
	Positive	Negative	Total	
QUANTA Flash Ro60				
Positive	80	0	80	Pos agreement = 96.4% (89.8–99.2%)
Negative	3	158	161	Neg agreement = 100.0% (97.7–100.0%)
Total	83	158	241	Total agreement = 98.8% (96.4–99.7%)
				*kappa* = 0.97 (95% CI 0.94–1.00)

**Table:** (c)

All patients (*n* = 241)	BioPlex 2200 SS-B			Percent agreement (95% confidence interval)
	Positive	Negative	Total	
QUANTA Flash SS-B				
Positive	26	3	29	Pos agreement = 60.5% (44.4–75.0%)
Negative	17	195	212	Neg agreement = 98.5% (95.6–99.7%)
Total	43	198	241	Total agreement = 91.7% (87.5–94.9%)
				*kappa* = 0.68 (95% CI 0.55–0.81)

**Table 3:** Overview of discrepant samples sorted according to disease state.

Sample ID	Diagnosis	QUANTRIX (cut-off = 6 U/mL)	D-tek Dot Blot	QUANTA Flash Ro52 (cut-off = 20 CU)	BioPlex 2200 (Ro52) (cut-off = 1.0)
ASX~0039	SjS	Not tested	1	30.2	0.2
ASX~0068	SjS	12	1	30.8	0.5
BEx0026	SjS	12	1	25	0.7
BEx0042	SjS	14	1	27.6	0.9
BEx0040	SLE	7	1	24.3	0.2
BEx0046	UCTD	10	1	56.4	0.4
BEx0058	UCTD	0	0	21.4	0.2
ASX~0088	RA	40	1	254.9	0.2
BSX0053	RA	Not tested	1	200.2	0.2
BSX0056	RA	Not tested	1	55.6	0.2
BSX0035	AT	Not tested	0	69.2	0.2

Sample ID	Diagnosis	QUANTRIX (cut-off = 6 U/mL)	D-tek Dot Blot	QUANTA Flash Ro60 (cut-off = 20 CU)	BioPlex 2200 (Ro60) (cut-off = 1.0)

BEx0006	UCTD	Not tested	0	4.9	8
BEx0051	UCTD	0	0	4.9	1.2
BEx0056	UCTD	0	0	8.2	1.1

Sample ID	Diagnosis	QUANTRIX (cut-off = 6 U/mL)	D-tek Dot Blot	QUANTA Flash SSB (cut-off = 20 CU)	BioPlex 2200 (SSB) (cut-off = 1.0)

BEx0001	SjS	0	0	8.4	3
BEx0014	SjS	0	0	24.8	0.9
BEx0035	SjS	0	0	13.1	8
BEx0036	SjS	Not tested	Not tested	25.5	0.2
ASX~0076	SLE	0	0	16.6	8
ASX~0082	SLE	0	0	8.5	8
BEx0054	SLE	0	0	3.3	1
BEx0013	UCTD	Not tested	0	4.2	8
BEx0022	UCTD	0	0	3.3	1.2
BEx0030	UCTD	Not tested	0	3.3	8
BEx0033	UCTD	Not tested	1	225.8	0.2
BEx0037	UCTD	0	0	5.3	1.9
BEx0058	UCTD	0	0	5.5	1.2
BEx0067	UCTD	0	0	5.1	4.7
BEx0068	UCTD	0	0	3.3	1.8
BEx0069	UCTD	0	0	3.3	2.7
ASX~0098	RA	0	0	3.3	1.2
BEx0060	RA	0	0	16	2.4
BSX0043	RA	0	0	3.3	4.2
BSX0091	HI	0	0	3.3	1.1

AT: atopic dermatitis; HI: healthy individual; UCTD: undifferentiated connective tissue disease; RA: rheumatoid arthritis; SjS: Sjögren syndrome; SLE: systemic lupus erythematosus.

**Table 4:** Multiple positivity for Ro60, Ro52, and SS-B.

BIO-FLASH	Single positive	Double positive	Triple positive	Ro60 > 110 CU	SS-B > 6.3 CU	SS-B > 482 CU
Sensitivity	75.9%	39.8%	21.3%	58.3%	37.0%	25.0%
Specificity	89.5%	96.2%	97.7%	94.7%	97.0%	97.0%
Likelihood ratio (+)	7.21	10.59	9.44	11.08	12.31	8.31
Likelihood ratio (−)	0.27	0.63	0.81	0.44	0.65	0.77

BioPlex 2200	Single positive	Double positive	Triple positive	Ro60 > 2.2 units	SS-B > 4.3 units	SS-B > 0.4 units

Sensitivity	80.6%	38.9%	21.3%	27.8%	25.0%	45.4%
Specificity	90.2%	96.2%	97.0%	96.2%	98.5%	95.5%
Likelihood ratio (+)	8.24	10.34	7.08	7.39	16.63	10.06
Likelihood ratio (−)	0.22	0.63	0.81	0.75	0.76	0.57

## Abbreviations

AT: Atopic dermatitis
ALBIA: Addressable laser bead assays
AMR: Analytical measuring range
CIA: Chemiluminescent immunoassay
CLSI: Clinical and laboratory standards institute
CTD: Connective tissue disease
LIA: Line immunoassays
MFI: Multiplex flow immunoassay
PM/DM: Polymyositis/dermatomyositis
RA: Rheumatoid arthritis
ROC: Receiver-operating characteristics
SARD: Systemic autoimmune rheumatic disease
SED: Suspected eye disease
SLE: Systemic lupus erythematosus
SjS: Sjögren's syndrome
SSc: Systemic sclerosis
UCTD: Undifferentiated connective tissue disease.

## References

[1] Mahler M, Fritzler MJ (2010). Epitope specificity and significance in systemic autoimmune diseases. *Annals of the New York Academy of Sciences*.

[2] Mahler M (2011). Sm peptides in differentiation of autoimmune diseases. *Advances in Clinical Chemistry*.

[3] Mahler M, Silverman ED, Schulte-Pelkum J, Fritzler MJ (2010). Anti-Scl-70 (topo-I) antibodies in SLE: myth or reality?. *Autoimmunity Reviews*.

[4] Schulte-Pelkum J, Fritzler M, Mahler M (2009). Latest update on the Ro/SS-A autoantibody system. *Autoimmunity Reviews*.

[5] Ghillani P, André C, Toly C (2011). Clinical significance of anti-Ro52 (TRIM21) antibodies non-associated with anti-SSA 60kDa antibodies: results of a multicentric study. *Autoimmunity Reviews*.

[6] Arbuckle MR, McClain MT, Rubertone MV (2003). Development of autoantibodies before the clinical onset of systemic lupus erythematosus. *The New England Journal of Medicine*.

[7] Ching KH, Burbelo PD, Tipton C (2012). Two major autoantibody clusters in systemic lupus erythematosus. *PLoS ONE*.

[8] López-Longo FJ, Rodríguez-Mahou M, Escalona-Monge M, González CM, Monteagudo I, Carreño-Pérez L (2003). Simultaneous identification of various antinuclear antibodies using an automated multiparameter line immunoassay system. *Lupus*.

[9] Fritzler MJ (2006). Advances and applications of multiplexed diagnostic technologies in autoimmune diseases. *Lupus*.

[10] Shovman O, Gilburd B, Barzilai O (2005). Evaluation of the BioPlex*™* 2200 ANA screen. Analysis of 510 healthy subjects: Incidence of natural/predictive autoantibodies. *Annals of the New York Academy of Sciences*.

[11] Shovman O, Gilburd B, Zandman-Goddard G, Yehiely A, Langevitz P, Shoenfeld Y (2005). Multiplexed AtheNA multi-lyte immunoassay for ANA screening in autoimmune diseases. *Autoimmunity*.

[12] Hanly JG, Su L, Farewell V, Fritzler MJ (2010). Comparison between multiplex assays for autoantibody detection in systemic lupus erythematosus. *Journal of Immunological Methods*.

[13] Robinson WH, DiGennaro C, Hueber W (2002). Autoantigen microarrays for multiplex characterization of autoantibody responses. *Nature Medicine*.

[14] Mahler M, Radice A, Yang W (2012). Development and performance evaluation of novel chemiluminescence assays for detection of anti-PR3 and anti-MPO antibodies. *Clinica Chimica Acta*.

[15] Bentow C, Swart A, Wu J (2013). Clinical performance evaluation of a novel rapid response chemiluminescent immunoassay for the detection of autoantibodies to extractable nuclear antigens. *Clinica Chimica Acta*.

[16] Op De Beéck K, Vermeersch P, Verschueren P (2012). Antinuclear antibody detection by automated multiplex immunoassay in untreated patients at the time of diagnosis. *Autoimmunity Reviews*.

[17] Mahler M, Fritzler MJ, Blüthner M (2005). Identification of a SmD3 epitope with a single symmetrical dimethylation of an arginine residue as a specific target of a subpopulation of anti-Sm antibodies. *Arthritis Research & Therapy*.

[18] Eisen MB, Spellman PT, Brown PO, Botstein D (1998). Cluster analysis and display of genome-wide expression patterns. *Proceedings of the National Academy of Sciences of the United States of America*.

[19] Chan EKL, Fritzler MJ, Wiik A (2007). AutoAbSC.Org—autoantibody standardization committee in 2006. *Autoimmunity Reviews*.

[20] Lee SA, Kahng J, Kim Y (2012). Comparative study of immunofluorescent antinuclear antibody test and line immunoassay detecting 15 specific autoantibodies in patients with systemic rheumatic disease. *Journal of Clinical Laboratory Analysis*.

[21] Hansson-Hamlin H, Rönnelid J (2010). Detection of antinuclear antibodies by the Inno-Lia ANA update test in canine systemic rheumatic disease. *Veterinary Clinical Pathology*.

[22] Copple SS, Martins TB, Masterson C, Joly E, Hill HR (2007). Comparison of three multiplex immunoassays for detection of antibodies to extractable nuclear antibodies using clinically defined sera. *Annals of the New York Academy of Sciences*.

[23] Avaniss-Aghajani E, Berzon S, Sarkissian A (2007). Clinical value of multiplexed bead-based immunoassays for detection of autoantibodies to nuclear antigens. *Clinical and Vaccine Immunology*.

[24] Fritzler MJ, Behmanesh F, Fritzler ML (2006). Analysis of human sera that are polyreactive in an addressable laser bead immunoassay. *Clinical Immunology*.

[25] Bayer PM, Bauerfeind S, Bienvenu J (1999). Multicenter evaluation study on a new HEp2 ANA screening enzyme immune assay. *Journal of Autoimmunity*.

[26] Bossuyt X (2000). Evaluation of two automated enzyme immunoassays for detection of antinuclear antibodies. *Clinical Chemistry and Laboratory Medicine*.

[27] Lehmann H-P, Fuhling I, Ott C, Hudepohl B, Haass M (2000). HEp2 ANA EIA: a new fully automated assay for the screening of antinuclear antibodies. *Israel Medical Association Journal*.

[28] Schmitt J, Papisch W (2002). Recombinant autoantigens. *Autoimmunity Reviews*.

